# Silver-Doped Titanium Oxide Layers for Improved Photocatalytic Activity and Antibacterial Properties of Titanium Implants

**DOI:** 10.3390/jfb15060163

**Published:** 2024-06-14

**Authors:** Aya Ali, Likhitha Polepalli, Sheetal Chowdhury, Mary A. Carr, Amol V. Janorkar, Mary E. Marquart, Jason A. Griggs, Joel D. Bumgardner, Michael D. Roach

**Affiliations:** 1 University of Mississippi Medical Center, Department of Biomedical Materials Science, Jackson, MS 39216, USA; ayagneady.1@gmail.com (A.A.); likhithapjo@gmail.com (L.P.); schowdhury@umc.edu (S.C.); ajanorkar@umc.edu (A.V.J.); jgriggs@umc.edu (J.A.G.); 2 University of Mississippi Medical Center, Department of Cell and Molecular Biology, School of Medicine, Jackson, MS 39216, USA; maryacarr3@outlook.com (M.A.C.); mmarquart@umc.edu (M.E.M.); 3 Department of Biomedical Engineering, University of Memphis, Memphis, TN 38152, USA; jbmdrgnr@memphis.edu

**Keywords:** anodization, titanium, antibacterial, photocatalytic activity, silver doping

## Abstract

Titanium has a long history of clinical use, but the naturally forming oxide is not ideal for bacterial resistance. Anodization processes can modify the crystallinity, surface topography, and surface chemistry of titanium oxides. Anatase, rutile, and mixed phase oxides are known to exhibit photocatalytic activity (PCA)-driven bacterial resistance under UVA irradiation. Silver additions are reported to enhance PCA and reduce bacterial attachment. This study investigated the effects of silver-doping additions to three established anodization processes. Silver doping showed no significant influence on oxide crystallinity, surface topography, or surface wettability. Oxides from a sulfuric acid anodization process exhibited significantly enhanced PCA after silver doping, but silver-doped oxides produced from phosphoric-acid-containing electrolytes did not. *Staphylococcus aureus* attachment was also assessed under dark and UVA-irradiated conditions on each oxide. Each oxide exhibited a photocatalytic antimicrobial effect as indicated by significantly decreased bacterial attachment under UVA irradiation compared to dark conditions. However, only the phosphorus-doped mixed anatase and rutile phase oxide exhibited an additional significant reduction in bacteria attachment under UVA irradiation as a result of silver doping. The antimicrobial success of this oxide was attributed to the combination of the mixed phase oxide and higher silver-doping uptake levels.

## 1. Introduction

Infection associated with medical devices, particularly bone implants, accounts for 26% of total infection associated with healthcare in the USA [1]. Up to 5% of fixation devices and prosthetic joints develop implant-associated infections [2]. Implant infections prolong patient recovery time and may lead to early implant failure [1,2,3,4]. The initial attachment of bacteria to implant surfaces has been shown to play a critical role in the rise of implant-associated infections [5].

Silver (Ag) has a centuries long history of use in medicine as an antimicrobial agent and is commonly utilized for treating burns, wounds, urinary tract infections, and central venous catheter infections [2,6,7,8]. Recently, adding silver particles or coatings to implant surfaces has become a strong research interest due to its killing efficiency against Gram-positive and Gram-negative bacteria as well as its good environmental stability [8,9,10,11,12].

Titanium implants have a long history of clinical use due to their excellent corrosion resistance, mechanical properties, and biocompatibility [13]. Titanium rapidly forms a thin amorphous oxide upon exposure to oxygenated environments. However, the un-modified titanium oxide surface is bioinert and presents less than ideal antibacterial behavior. Electrochemical anodization is commonly used to modify titanium oxide surfaces to form crystalline oxides, add beneficial surface chemistry, and produce micro- and nano-scaled surface topographies [14,15,16,17]. Furthermore, crystalline titanium oxides are known to exhibit photocatalytic activity (PCA) when exposed to ultraviolet light of sufficient energy [18,19,20,21]. In the photocatalytic reaction, irradiation of titanium oxide with UVA light causes electrons to jump from the valence band to the conduction band. This process forms electron–hole pairs which can react with available oxygen and water to form reactive oxygen species (ROS) [18,21,22,23,24]. The UVA-generated ROS have been reported to decompose organic molecules and compounds which can lead to bacterial cell death [21,22].

Doping titanium oxides with beneficial chemistries has been shown to alter the PCA and antimicrobial responses [24,25,26,27,28,29,30]. Phosphorus doping has been shown to increase the PCA of anatase or mixed phase oxides when compared to non-phosphorus-containing titanium oxide counterparts [31,32]. Previous studies in our laboratories developed mixed-acid anodization electrolytes to form phosphorus-doped crystalline oxides on titanium implant substrates [3,15,26]. Both anatase phase and mixed anatase and rutile phase phosphorus-doped oxides exhibited approximately 50% killing efficacy against *Streptococcus sanguinis* following a 15 min pre-illumination with a 365 nm UVA light [3]. Silver-doped anatase phase oxide spheres have also been shown to exhibit higher PCA compared to undoped control oxide spheres [30]. Titanium oxide nanotubes with deposited silver nanoparticles showed significantly reduced numbers of attached *S. sanguinis* and *Lactobacillus salivarius* compared to anodized titanium controls and unmodified titanium [33].

The present study investigated the effects of silver doping additions to three established anodization processes in an attempt to enhance the PCA and antibacterial responses. Two of these anodization processes produced phosphorus-doped oxides due to phosphoric acid being an electrolyte component. The third anodization process involved a 1M sulfuric acid electrolyte that has been commonly used in other titanium anodization studies [34,35,36]. The primary study objective was to assess the benefits of silver-doping additions to established anodization processes that have already been shown to exhibit beneficial PCA and bacterial responses. A secondary objective was to identify any differences in the oxide surface properties of the silver-doped oxides compared to their counterpart oxides without silver.

## 2. Materials and Methods

### 2.1. Sample Preparation

Commercially pure titanium grade 4 (CPTi) disc specimens with a 3 mm thickness and 12.7 mm diameter were cut from a centerless ground bar stock, cleaned in a laboratory detergent solution in an ultrasonic bath, and rinsed with distilled water. Anodization was performed in potentiostatic mode using a DC rectifier (300 V, 10 A, Dynatronix, Amery, WI, USA) with two CPTi strip counter electrodes. A stepped waveform consisting of 12 V, 10 s steps, was applied up to a final forming voltage of 180 V in 500 mL of each electrolyte listed in Table 1. The total duration of the applied anodization waveform was 150 s. Three anodization electrolytes (A, B, and C) consisted of mixtures of sulfuric acid (ACS, Fisher Scientific, Waltham, MA, USA), phosphoric acid (ACS, Fisher Scientific, Waltham, MA USA), hydrogen peroxide (30%, Fisher Scientific, Waltham, MA, USA), and oxalic acid (ACS, Al-fa Aesar, Haverhill, MA, USA) components. Electrolytes A and B have been previously documented to produce phosphorus-doped anatase phase and mixed phase oxides on commercially pure titanium substrates [3,15,26]. Electrolyte C is 1 M sulfuric acid, which has been commonly used in other titanium anodization studies [34,35,36]. Silver nitrate (99.85%, Thermo Fisher Scientific, Waltham, MA, USA) was added to its saturation limit in each of these electrolytes to create the remaining A + Ag, B + Ag, and C + Ag electrolytes (Table 1) used in this study.

### 2.2. Oxide Layer Characterization

Thin-film X-ray diffraction (XRD) (Scintag Inc., Waltham, MA, USA, XDS2000) was used to determine the crystalline phases within each oxide. Anodized specimens were rotated 1° away from the copper X-ray source (1.54 A° Cu-Kα) and the XRD scans were conducted over two-theta angles ranging from 24° to 30° using a continuous scan rate of 2°/min. The surface topographies of representative specimens (n = 3) from each oxide were examined using scanning electron microscopy (SEM) (Supra 40, Zeiss, Oberkochen, Germany) at a 3 kV acceleration voltage. The surface roughness of representative specimens (n = 3) from each oxide was evaluated using atomic force microscopy (AFM, Bruker, Santa Barbara, CA, USA, Bioscope Catalyst) in ScanAssyst mode (0.592 Hz, and 512 samples/line). Gwyddion software (Version 2.58, Department of Nanometrology, Czech Metrology Institute, Okružní Brno, Czechia) was used to calculate the roughness average (R_a_) and the average maximum height roughness (R_z_). The surface wettability of each oxide was also determined by measuring the water contact angles on the surface of representative specimens from each group (n = 3) using the sessile drop technique with a 3 µL droplet size. The surface chemistry and the electronic state of the surface atoms within each oxide were determined using X-ray photoelectron spectroscopy (XPS) on a K-alpha XPS system (Thermo Fisher Scientific Instruments, Waltham, MA, USA). For the XPS studies, a monochromatic X-ray source at 1486.6 eV, corresponding to the Al Kα line and X-ray power of 75 W at 12 kV, was used for all specimens with a spot size of 400 µm^2^. High-resolution spectra for Ti 2p, O 1s, P 2p, and Ag 3d were collected at a 40 eV pass energy, a step size of 0.1 eV, and 50 scans. XPS data analyses were performed using the manufacturer’s software (Advantage 5.9911 Surface Chemical Analysis, ThermoFisher Scientific, Waltham, MA, USA).

In order to examine the oxide thickness values from each anodization process, representative cross-sectional specimens from each oxide were prepared and polished. Triplicate images from each oxide cross-section were acquired using SEM. Five thickness measurements were performed on each image, for a total of fifteen thickness measurements per oxide. Electron backscattered diffraction (EBSD, OIM Analysis 8.0, EDAX, Pleasanton, CA, USA) analyses were also performed on the oxide cross-sections to characterize the phase fractions and the spatial distributions of the oxide phases in each group. For EBSD analyses, the polished cross-sectional specimens were tilted to 70° and scanned using an accelerating voltage of 12 kV.

### 2.3. Photocatalytic Degradation of Methylene Blue

The photocatalytic activity of each oxide was evaluated using a methylene blue (MB) degradation assay using a 1% aqueous MB solution. Specimens from each oxide were soaked in 2 mL of the MB solution (LabChem, Zelienope, PA, USA) for a period of 16 h to saturate the porous oxide surfaces and prevent false MB degradation readings. After soaking, the original MB solution was removed and replaced with 2 mL of fresh new MB solution. Twenty-four-well cell culture plates containing representative oxide specimens (n = 6) were exposed to ultraviolet (UVA) illumination (365 nm peak wavelength, 8 mW/cm^2^) for a period of 4 h. Measurements were taken from the 16 h pre-soak solution and from the UVA-irradiated solutions at 0, 30, 60, 90, 120, 180, and 240 min timepoints. Three 50 µL aliquots of the MB solution from the wells containing each oxide specimen were then transferred to a 96-well plate. An ELX800 Universal Microplate Reader (BioTek Instruments, Winooski, VT, USA) was used to measure the absorbance of the MB aliquots from each oxide specimen at 660 nm. The photocatalytic activity percentage was then calculated using the following equation:

Photocatalytic activity (%) = [(c_0_ − c)/c_0_] × [c_1_/c_0_] × 100 (1)

where c_0_ is the concentration of the MB solution before UV illumination, c is the concentration of the MB solution after UV illumination at each of the timepoints, and c_1_ is the concentration of the MB solution after the 16 h pre-soak.

### 2.4. Bacterial Testing

The number of bacteria attached to the surfaces of each oxide with and without UVA illumination was evaluated using a colony-forming unit (CFU) counting technique that has been used in previous studies [26,37]. Frozen aliquots of *Staphylococcus aureus* strain 11-14697 (kindly provided by Darlene Miller, Bascom Palmer Eye Institute, Miami, FL, USA) were streaked onto tryptic soy agar (TSA) and incubated at 37 °C overnight. A single colony from the TSA was then used to inoculate 10 mL of tryptic soy broth (TSB) and incubated for 16–20 h at 37 °C with shaking. The bacteria were diluted 1:100 in 20 mL of sterile TSB and grown to OD600, yielding a concentration of 10^8^ colony-forming units per mL. The bacteria were then centrifuged at 2025× *g* RCF for 30 min and the resulting pellet was suspended in 20 mL of phosphate buffered saline (PBS). Anodized specimens of each oxide (n = 8) were placed in 24-well cell culture plates. Then, 1 mL of bacteria at a concentration of 1 × 10^8^ CFU/mL was added to each well. The specimens were then incubated at room temperature for a period of 4 h. Half of the specimens were then illuminated by UVA, while the other half were covered with foil and kept under dark conditions for the 4 h period. After 4 h, the specimens were washed twice with PBS and 1 mL of fresh sterile PBS was added to each specimen followed by sonication to remove attached bacteria. The released bacteria were then serially diluted and plated on TSA and incubated overnight at 37 °C. On the following day, colony-forming units (CFU) were counted and CFU/mL levels were calculated.

### 2.5. Pre-Osteoblast Culture

Cytocompatibility testing was only performed on the three silver-doped oxides (A + Ag, B + Ag, and C + Ag) and a non-modified CPTi surface control group (NT). Mouse pre-osteoblastic cells (MC3T3-E1, Subclone 4; American Type Culture Collection, Manassas, VA, USA) were cultured and maintained in alpha modified Eagle’s minimum essential medium with 10% fetal bovine serum and 1% penicillin–streptomycin at 37 °C and 5% CO_2_. Specimens from each oxide (n = 3) were autoclaved and seeded with approximately 50,000 cells/cm^2^. The media were changed every 48 h for up to 7 days. Cell viability of silver-doped groups was assessed using a live/dead TM viability/cytotoxicity assay kit (Invitrogen, Carlsbad, CA, USA) on representative specimens (n = 3) of each oxide on day 7. Images were captured using an epifluorescence microscope (IX81, Olympus, Hachioji, Tokyo, Japan) with image analysis software (Slidebook 4.2.0.10, Olympus, Center Valley, PA, USA).

For cell viability quantification, an MTT assay was conducted on days 7 and 14 for the A + Ag, B + Ag, and C + Ag oxide groups and the NT CPTi control group (n = 3) as described previously [38]. On days 7 and 14, the medium was removed, replaced with MTT solution (Life Technologies Corporation, Eugene, OR, USA), and incubated at 37 °C for 4 h. Wells with cell culture medium and MTT solution but no pre-osteoblastic cells present were used as controls. After a 4 h incubation period, the medium was removed and DMSO (Sigma Aldrich, St. Louis, MO, USA) was added and incubated for 10 min. The contents for each well were collected, and three replicates of each oxide group were then plated in a 96-well plate and read using an ELx800 plate reader (Biotek, Winooski, VT, USA) at 540 nm.

### 2.6. Statistical Analyses

Two-way ANOVAs (α = 0.05) were utilized to determine the effect of the main factors of the oxide group and silver doping on surface roughness, water contact angles, oxide layer thickness values, and MB degradation percentages. If significant differences were shown for these main factors or their interaction, a Tukey *post hoc* analysis was used to determine differences between individual oxide groups. To evaluate differences in the phosphorus and silver-doping uptake levels in the oxide groups, one-way ANOVAs (α = 0.05) were utilized to analyze the data collected in the XPS analyses. If significant differences were shown, a Tukey *post hoc* analysis was used to determine differences between individual oxide groups. A three-way ANOVA (α = 0.05) was utilized to determine significant differences in *S. aureus* attachment to the oxide surfaces due to the main factors including oxide group, silver doping, and the light condition (dark or UVA-illuminated conditions). If significant differences were shown for these main factors or their respective interactions, a Tukey *post hoc* analysis was used to determine differences between individual oxide groups. Finally, differences in cell viability were evaluated using one-way ANOVAs (α = 0.05) on days 7 and 14. If significant differences were shown, a Tukey *post hoc* analysis was used to compare differences between individual oxide groups.

## 3. Results

### 3.1. Oxide Layer Characterization

**Figure 1:**
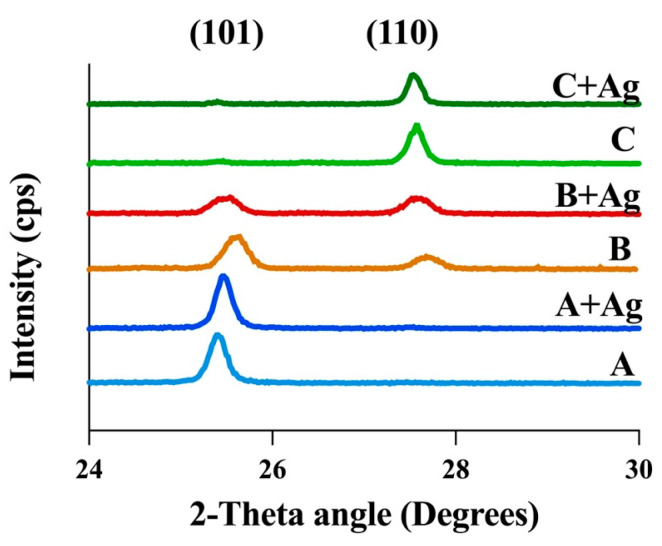
Representative XRD scans are shown for each oxide group. Anatase (101) and rutile (110) diffraction peaks are shown at two-theta angles of 25.4° and 27.6°, respectively.

Representative XRD scans for each oxide group are shown in Figure 1. Oxide groups A and A + Ag exhibited primarily anatase phase oxides as shown by the peak centered at 25.4°. Oxide groups B and B + Ag revealed mixed phase oxide formation, consisting of both anatase and rutile phases, as indicated by peaks centered at 25.4° and 27.6°, respectively. Oxide groups C and C + Ag exhibited primarily rutile phase oxides as shown by the peak centered at 27.6°.

**Figure 2:**
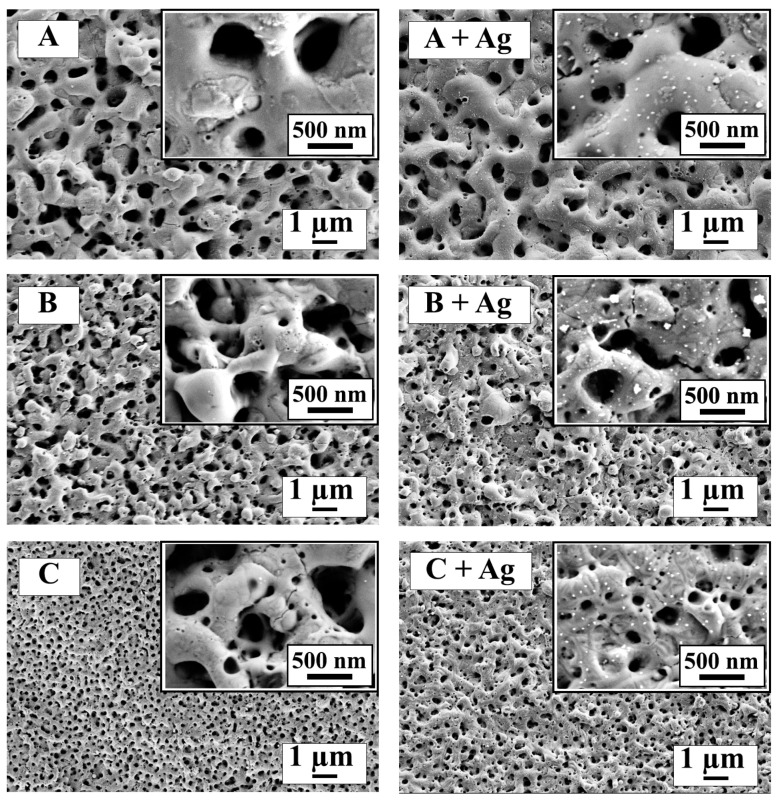
Representative SEM images are provided to show the surface topographies for each oxide group. The larger background images for each oxide were acquired at 5000× magnification in order to examine larger surface areas. The inset images in the top right corner of each frame were acquired at 60,000× magnification in order to assess the presence, or absence, of nano-sized silver-containing particles.

Representative SEM surface topography images are shown for each oxide at two different magnifications in Figure 2. The surface topographies exhibited a relatively uniform distribution of submicron porosity for each anodized oxide in the study. Higher-magnification inset images of the silver-doped oxides revealed nano-sized silver particles on the surfaces which were absent in the counterpart oxides without silver doping. The nano-sized oxide particles on B + Ag oxide appeared larger than those formed on the A + Ag and C + Ag oxides.

**Figure 3:**
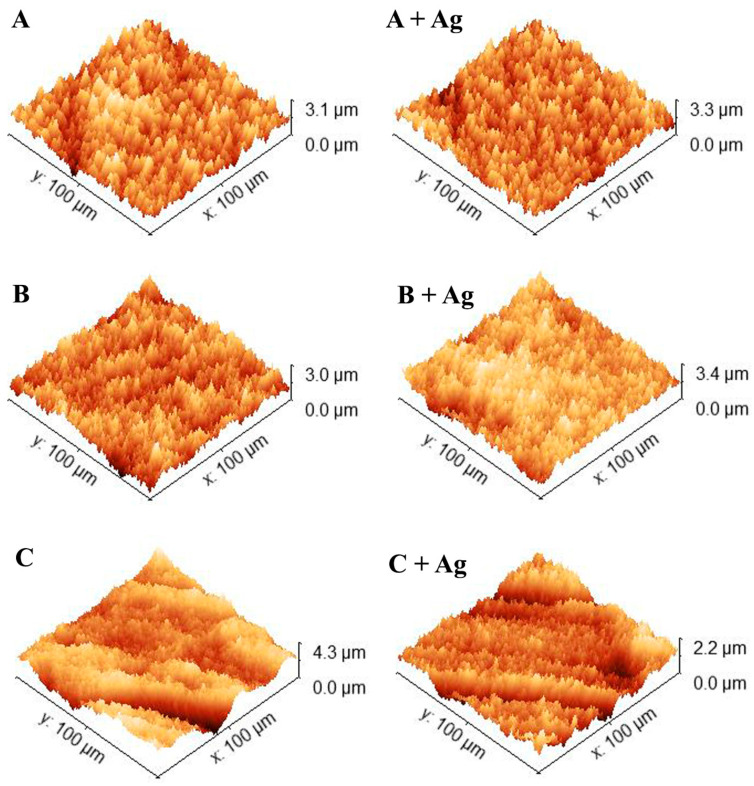
Representative 100 × 100 µm^2^ 3D AFM scans are shown for each oxide group.

Figure 3 shows representative 100 µm × 100 µm 3D AFM scans for each oxide. The average surface roughness (R_a_) and the average maximum height roughness (R_z_) for representative scans (n = 3) are compiled in Table 2. A two-way ANOVA revealed no significant interaction between the main effects of oxide group or silver doping on oxide R_a_ (*p* = 0.15). Subsequent simple main effects analyses showed that neither oxide group (*p* = 0.22) nor silver doping (*p* = 0.21) showed a significant effect on oxide R_a_. A two-way ANOVA also revealed no significant interaction between the effects of oxide group or silver doping on oxide R_z_ (*p* = 0.14). Subsequent simple main effects analyses showed that neither oxide group (*p* = 0.75) nor silver doping (*p* = 0.15) showed a significant effect on oxide R_z_.

**Figure 4:**
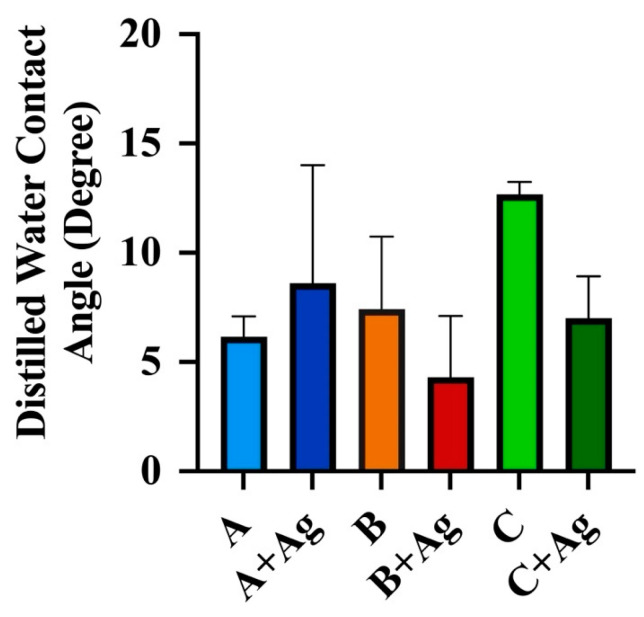
Water contact angles are shown for each oxide group.

The water contact angle measurements for each oxide group are provided in Figure 4. All oxide groups exhibited contact angles less than 90 °C, indicative of hydrophilic surfaces. A two-way ANOVA revealed no significant interaction between the effects of oxide group or silver doping on the surface contact wetting angles (*p* = 0.09). Subsequent simple main effects analyses showed that neither oxide group (*p* = 0.11) nor silver doping (*p* = 0.16) exhibited a significant effect on the contact wetting angle.

**Figure 5:**
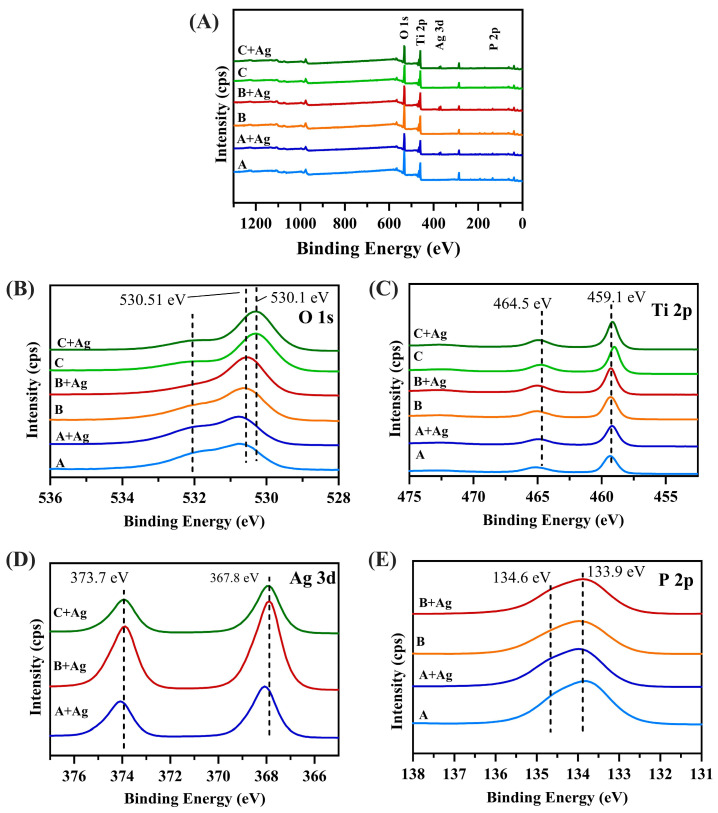
(**A**) Representative XPS survey spectra are shown for each oxide group. Representative high-resolution XPS spectra of (**B**) Ti 2p, (**C**) O 1s, (**D**) Ag 3d, and (**E**) P 2p are shown for the respective oxide groups.

Representative survey and high-resolution XPS spectra of Ti 2p, O 1s, S 2p, N 1s, and C1s for each oxide are compiled in Figure 5. The surface chemistries of each oxide group are compiled from the XPS datasets in Table 3. The Ti 2p spectra in Figure 5B revealed the presence of titanium at the highest oxidation state (Ti4^+^), indicating the formation of titanium oxide, at approximately 458.8 eV and 465 eV for all oxide groups [26,39]. O 1s spectra, as shown in Figure 5C, revealed the presence of two main peaks at 530.1 eV and 532.1 eV, which indicated the formation of titanium oxide and hydroxyl groups, respectively [26,39]. An additional O 1s peak was present at 532 eV for each oxide, which indicated the presence of additional hydroxyl groups and phosphates [26]. Oxide groups A and A + Ag exhibited the highest intensities for this additional O 1s peak. This finding was not surprising, as these oxide groups contained the highest phosphoric acid concentration in the anodization electrolyte (Table 1). Figure 5D shows the high-resolution spectra for Ag 3d in each oxide. The three silver-doped oxide groups exhibited two peaks at approximately 367.8 eV and 373.7 eV. This finding indicated the presence of Ag+ and the formation of Ag_2_O [33,40,41]. A one-way ANOVA on the silver-doping uptake levels found the B + Ag oxide to exhibit a significantly higher amount of silver doping (1.28 ± 0.05 at %) compared to the A + Ag oxide group (0.80 ± 0.09 at %) (*p* = 0.0001) and the C + Ag oxide group (0.61 ± 0.03 at %) (*p* < 0.0001). In addition, silver-doping levels in the A + Ag oxide group were also shown to be significantly higher than those for the C + Ag oxide group (*p* = 0.016). The P 2p spectra for the phosphorus-doped oxides (A, A + Ag, B, and B + Ag), as shown in Figure 5E, revealed a main peak at 133.9 eV which represents the formation of TiPO_4_ and a peak shoulder at 134.6 eV, indicating the formation of P_2_O_5_ [26]. A one-way ANOVA on the phosphorus-doping uptake levels found the A oxide to exhibit a significantly higher amount of phosphorus doping (3.64 ± 0.12 at %) compared to the B oxide group (2.73 ± 0.11 at %) (*p* = 0.0005) and the A + Ag oxide to exhibit a significantly higher amount of phosphorus doping (3.14 ± 0.23 at %) compared to the B + Ag oxide group (2.65 ± 0.15 at %) (*p* < 0.02). Interestingly, the A + Ag oxide showed a significantly lower phosphorus uptake compared to its counterpart A oxide without silver doping (*p* = 0.02). However, the B + Ag and B oxide groups exhibited statistically similar phosphorus-doping levels (*p* = 0.93).

**Figure 6:**
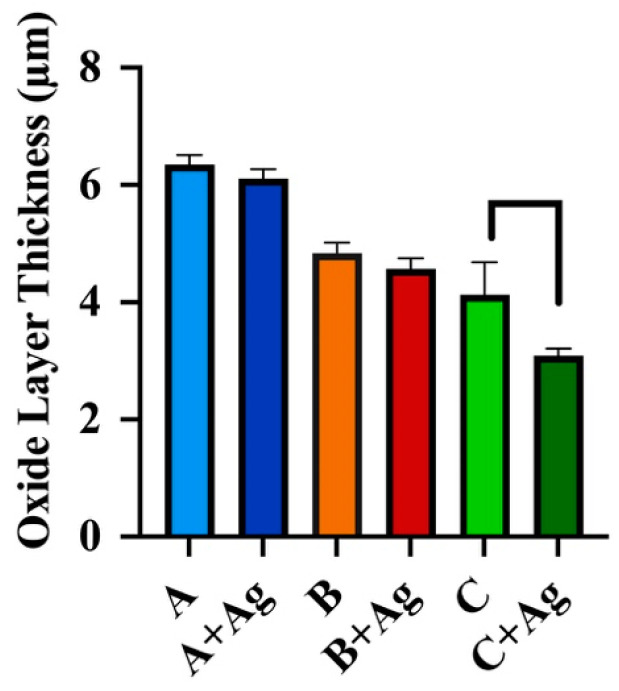
Oxide layer thickness values are compiled for each oxide group. The C + Ag oxide was shown to exhibit significantly lower thickness compared to its C oxide counterpart without silver doping (*p* = 0.005).

Oxide layer thickness values for each oxide group, measured from the cross-sectional SEM images, are compiled in Figure 6. A two-way analysis revealed a significant interaction between the effects of oxide group and silver doping on the oxide layer thickness values (*p* = 0.039). Subsequent simple main effects analyses showed that oxide group (*p* < 0.0001) and silver doping (*p* = 0.002) each had a statistically significant effect on the oxide thickness. A Tukey *post hoc* analysis showed significant reductions in thickness between the C + Ag oxide group and the counterpart C oxide group without silver doping (*p* = 0.005).

**Figure 7:**
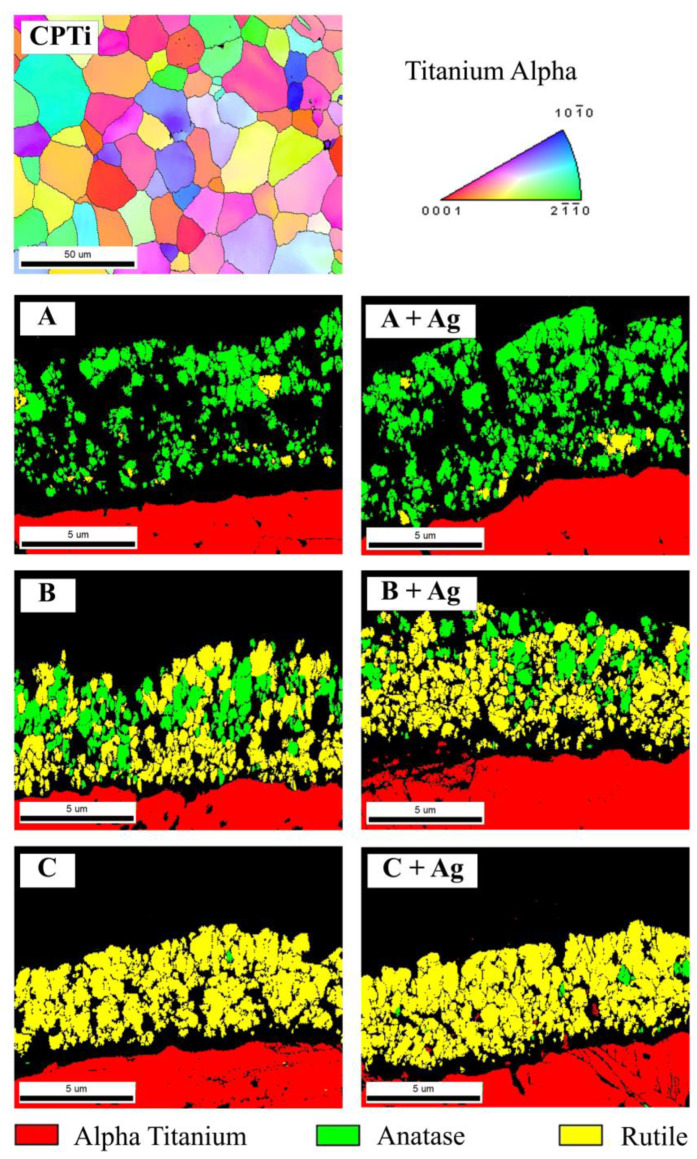
(**Top**) A representative EBSD grain orientation map is provided for the titanium substrate material. A color-coded inverse pole figure legend depicting the grain orientations for hexagonal close-packed alpha phase titanium is also included to the right of the map. (**Bottom**) Representative cross-sectional EBSD phase maps are provided for each oxide group.

Figure 7 compiles the cross-sectional EBSD analyses for the substrate CPTi material and the anodized oxide groups. A representative grain orientation map for the CPTi substrate material is shown at the top of Figure 7. A grain size analysis revealed an average grain diameter of approximately 10 µm for the titanium material. EBSD phase maps of the cross-sections from each oxide group are shown at the bottom of Figure 7. Oxide groups A and A + Ag revealed primarily anatase phase oxides with a few rutile grains near the interface with the CPTi substrate material. This result was consistent with the anatase phase peaks shown for these oxide groups in the bulk XRD phase analysis shown in Figure 1. Oxide groups B and B + Ag exhibited an intermingled mixture of anatase and rutile phase areas, which was also in agreement with the bulk XRD findings shown in Figure 1. More importantly, the anatase and rutile phase areas within the B and B + Ag oxide cross-sections were shown to be in close proximity to the outermost oxide surfaces. Thus, the EBSD analysis gives us additional spatial information on the localized oxide phase distributions within the mixed phase oxides that is not discernable from the XRD datasets. Oxide groups C and C + Ag showed primarily rutile phase oxides with a few anatase grains dispersed throughout the oxide cross-sections, which also agreed with the bulk XRD findings in Figure 1.

### 3.2. Photocatalytic Degradation of Methylene Blue

**Figure 8:**
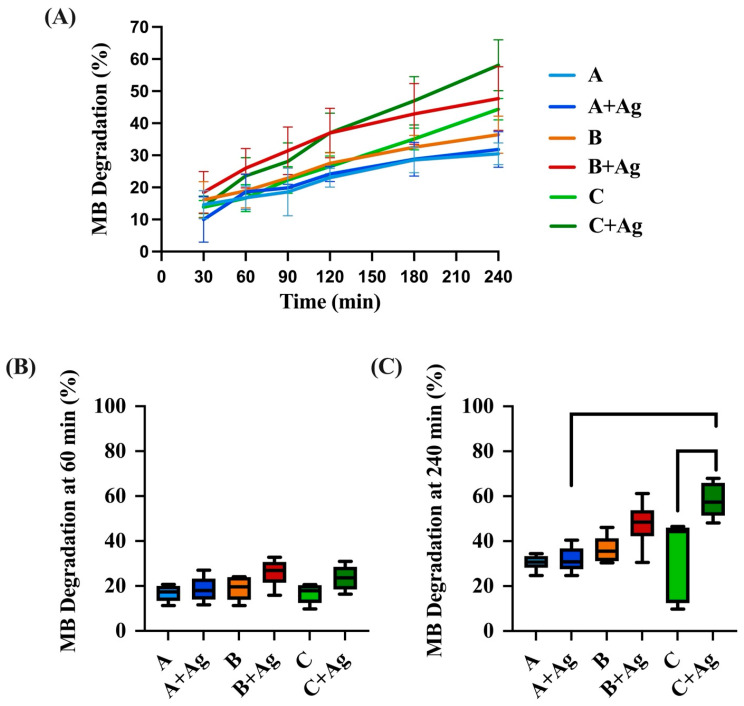
(**A**) The relative methylene blue degradation levels for each anodized oxide area shown as a function of UVA irradiation time. (**B**) The relative methylene blue degradation levels after 60 min of UVA irradiation are compiled for each oxide. (**C**) The relative methylene blue degradation levels after the 240 min of UVA irradiation are compiled for each oxide. Brackets represent significant differences between oxide groups. Significantly higher methylene blue degradation levels were shown for the C + Ag oxide group compared to the C oxide group (*p* = 0.0017). The C + Ag oxide also exhibited significantly higher degradation levels compared to the A + Ag oxide group (*p* = 0.0006).

The relative photocatalytic activity of each oxide group was determined by a methylene blue degradation assay and is compiled in Figure 8. After 60 min of UVA illumination, a two-way ANOVA revealed no significant interaction between the effects of oxide group and silver doping on the MB degradation shown for the anodized oxides (*p* = 0.38). A subsequent simple main effects analysis revealed silver doping to have a statistically significant effect (*p* = 0.005) on MB degradation. However, a Tukey *post hoc* analysis revealed no significant differences in MB degradation between any of the individual oxide groups. The other simple main effects analysis revealed the oxide group (*p* = 0.096) did not have a significant effect on MB degradation.

After 240 min, a two-way ANOVA did reveal a significant interaction between the effects of oxide group and silver doping on the MB degradation shown for the anodized oxides (*p* = 0.02). Subsequent simple main effects analyses showed both the oxide group and silver doping exhibited significant effects on MB degradation (*p* < 0.001 and *p* = 0.002, respectively). Furthermore, a *post hoc* Tukey analysis revealed the C + Ag oxide exhibited significantly higher MB degradation compared to the counterpart C oxide without the silver doping (*p* = 0.0017) as shown in Figure 8C. However, no significant differences were shown between the A and A + Ag oxide groups (*p* = 0.99) or the B and B + Ag oxide groups (*p* = 0.34). Among the silver-doped oxides, the C + Ag oxide also exhibited significantly higher MB degradation compared to A + Ag oxide (*p* = 0.0006).

### 3.3. Bacterial Testing

**Figure 9:**
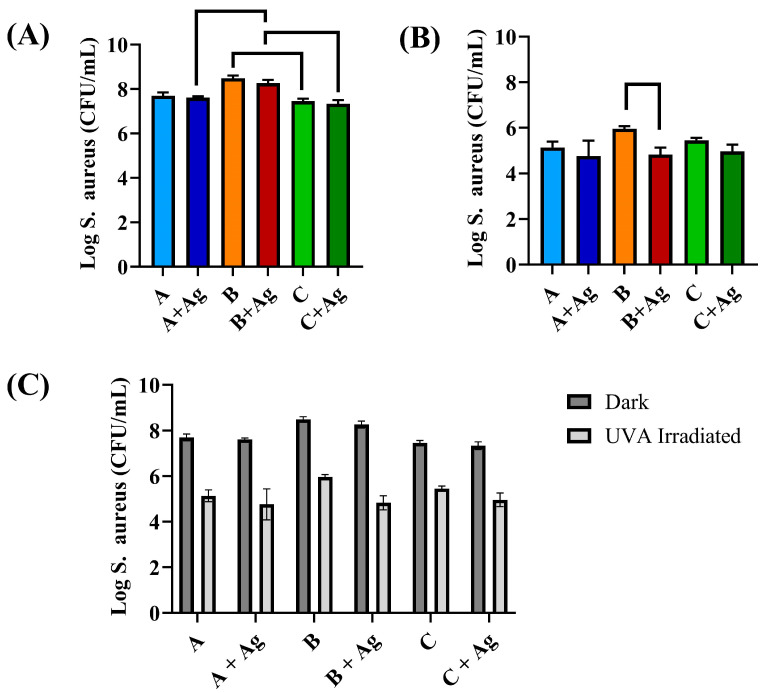
A comparison of the number of attached *S. aureus* is shown for each oxide group after 4 h under dark and UVA-light-irradiated conditions. (**A**) A comparison of the relative bacterial attachment to each oxide under dark conditions is provided. Brackets represent significant differences shown in bacterial attachment under dark conditions. Significantly decreased bacterial attachment levels were shown for the A + Ag and C + Ag oxides compared to the B + Ag oxides. For the oxides that did not contain silver, significantly decreased bacterial attachment was also shown for the C oxide compared to the B oxide. (**B**) A comparison of the relative bacterial attachment under UVA-irradiated conditions is provided. Brackets represent significant differences in bacterial attachment levels shown for the B + Ag oxide compared to its counterpart B oxide that did not contain silver doping. (**C**) A direct comparison of the relative bacterial attachment to each oxide under dark and UVA-irradiated conditions is provided. Each oxide showed significantly decreased bacterial attachment under UVA-irradiated conditions compared to dark conditions.

Figure 9 shows the relative *S. aureus* attachment to each of the oxide surfaces after 4 h under dark (e.g., without UVA) and light (direct UVA irradiation) conditions. A three-way ANOVA revealed there was not a significant three-way interaction between oxide group, silver doping, and the light conditions (*p* = 0.21). However, there were significant two-way interactions between the oxide group and light condition, oxide group and silver doping, and silver doping and light condition (*p* = 0.05, 0.0006, and 0.002, respectively). Simple main effects analyses showed the oxide group, light condition, and silver doping to each have a significant effect on bacterial attachment on the oxide surfaces (*p* < 0.0001). A *post hoc* Tukey analysis revealed no significant difference between the silver-doped and non-silver-containing oxide counterparts for a given oxide under dark conditions. For the silver-doped oxide groups under dark conditions, the A + Ag and C + Ag oxide groups showed significantly fewer attached bacteria compared to the B + Ag oxide group (*p* = 0.008 and 0.0002, respectively) as shown in Figure 9A. For the non-silver-containing oxides under dark conditions, the B oxide showed significantly higher numbers of attached bacteria compared to the C oxide (*p* = 0.001).

Under UVA-irradiated light conditions, the B oxide showed a significantly higher number of attached bacteria compared its silver-doped B + Ag counterpart oxide (*p* < 0.0001) as shown in Figure 9B. This difference was attributed to the silver-doping additions in the B + Ag oxide. No significant differences were shown between the A and A + Ag or C and C + Ag oxide groups. Finally, a direct comparison or the relative bacterial attachment under dark and UVA-irradiated light conditions is shown in Figure 9C. Each oxide group was shown to exhibit some photocatalytic bactericidal effect on the *S. aureus* as significantly lower attachment was shown under UVA-irradiated conditions compared to dark conditions (*p* < 0.0001).

### 3.4. Pre-Osteoblast Testing

**Figure 10:**
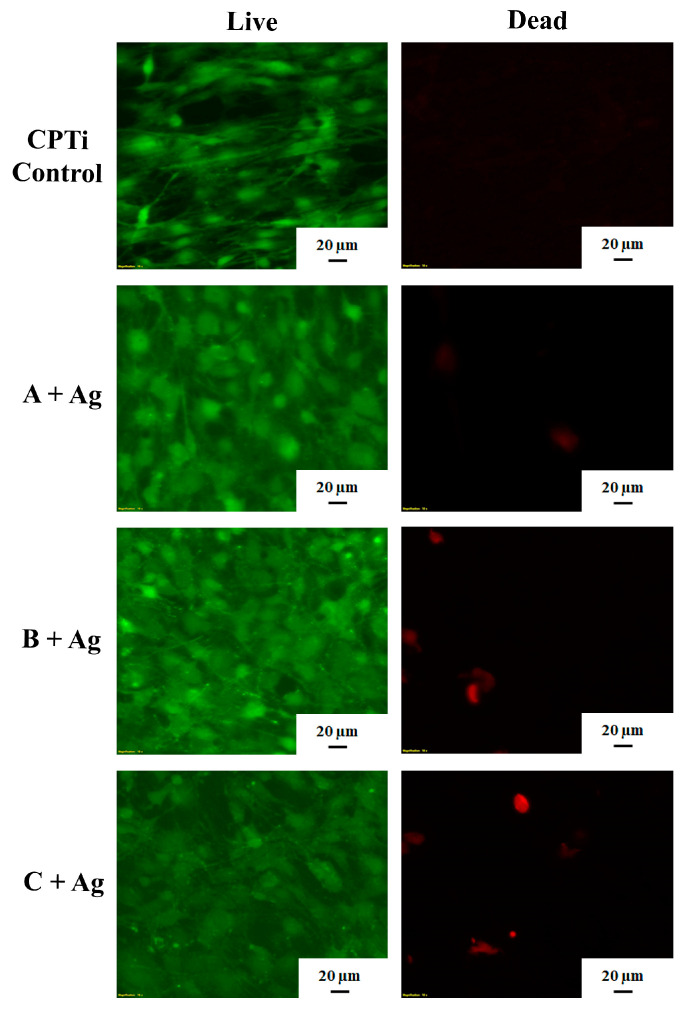
Representative live/dead stained MC3T3 osteoblast images are shown for day 7 for each group. Confluent live cell distributions are shown in green in the left images for each oxide. A sparse distribution of dead cells is shown in red in the right images for each oxide.

**Figure 11:**
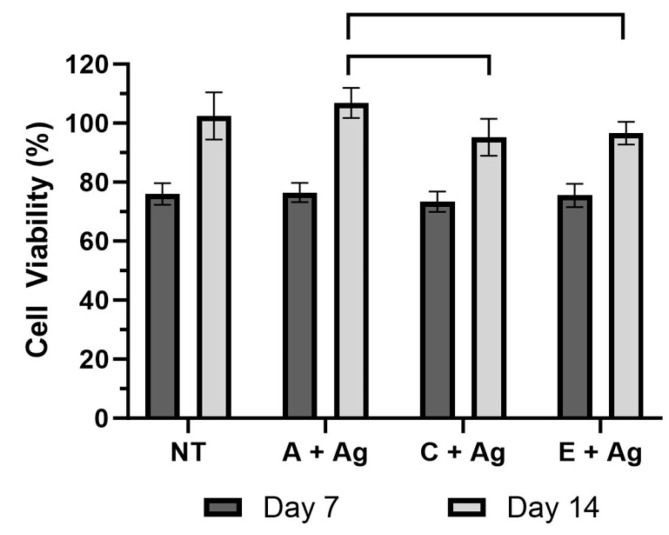
The MTT assay cell viability results from days 7 and 14 are compiled for each oxide. Brackets in this figure represent statistically significant differences in cell viability shown between the oxide groups.

Figure 10 shows the results of the live/dead assay indicating a high number of live cells and very few dead cells present on the surfaces of each oxide group. The results of the MTT assay (Figure 11) revealed no significant differences in cell viability for any of the groups tested on day 7 (*p* = 0.284). There were no significant differences between the non-modified CPTi control surface group and any of silver-doped oxide groups on day 14. However, the A + Ag oxide showed significantly higher viability values compared to the B + Ag and C + Ag oxides (*p* = 0.0014 and 0.0051, respectively) on day 14.

## 4. Discussion

Bone implant-associated infections can lead to loss of supporting bone and implant failure [42]. Antibiotic treatments are often ineffective in treating such infections due to the formation of biofilms [12]. Since the naturally forming oxide on titanium implants lacks inherent antibacterial properties, surface modifications that may reduce bacterial attachment, and subsequent infections, are needed. Anodization processes provide at least two strategies to improve the bacterial response of titanium oxide surfaces. The first strategy involves converting the naturally forming amorphous titanium oxides into anatase or rutile crystalline phases that possess photocatalytic properties. Subsequent UVA light irradiation of these crystalline oxide phases has been shown to generate reactive oxygen species in oxygenated environments which attack bacterial cell membranes, leading to their death [23,24]. The second strategy involves the addition of beneficial non-metal or metal chemical dopants to the titanium oxide layers to improve the photocatalytic and antimicrobial activities of the oxides [24,26,27,28,29,43]. Previous studies have demonstrated improved photocatalytic activity after doping titanium oxide with phosphorus [25,26,31] or silver [30,43]. The deposition of silver onto titanium surfaces has been previously shown to inhibit the growth of *S. aureus* [9,44]. In the present study, we combined these two strategies to reduce the bacterial attachment on anodized titanium. Specifically, silver doping was added to three anodization processes that previously showed good photocatalytic activity results.

The SEM, AFM, and surface wettability results revealed that silver doping did not significantly change the surface topography, porosity, or the wettability of the oxides compared to their counterparts without silver. However, SEM images did show nanoparticles on the surfaces of each silver-doped oxide, which were not present on the non-silver-containing groups. Previous titanium oxide studies utilizing silver doping or silver deposition have reported similar silver-containing nanoparticles to be present on the surfaces [33,44,45]. The XPS analyses in the present study confirmed the presence of silver-doping in the A + Ag, B + Ag, and C + Ag oxides. Interestingly, the B + Ag oxide revealed the highest XPS atomic percentage of silver doping, despite containing the lowest concentration of silver nitrate within the anodization process electrolytes listed in Table 1. The nano-sized silver-containing particles shown in the Figure 2 SEM images for the B + Ag oxide also appeared larger than those for the A + Ag and C + Ag oxides. It is worth noting again that silver nitrate was dissolved to its saturation limit in each of the previously successful electrolytes in this study. Silver nitrate was chosen as the salt for silver doping in this study, since it has been widely used in other silver-doping coating studies [6,9,33].

The XRD and EBSD analyses showed no significant changes to the crystalline structures of the silver-doped oxides compared to their counterparts without silver. The A and A + Ag oxide groups exhibited mainly anatase phase oxide layers as a result of the higher phosphorus-doping levels shown in the XPS dataset in Table 3. A number of previous anodization studies have shown that phosphorus doping stabilizes the anatase phase and restrains the anatase-to-rutile phase transformation [46,47,48]. The C and C + Ag oxides in the present study, with no phosphoric acid additions in the anodization process electrolytes, showed almost complete transformation to rutile phase oxides. However, the B and B + Ag oxide groups, which had lower concentrations of phosphoric acid in the electrolyte and lower levels of phosphorus uptake as shown in the XPS dataset in Table 3, formed mixed phase oxides containing intermingled anatase and rutile crystalline phases. EBSD analyses further revealed that the anatase and rutile phase distributions within the B and B + Ag oxides were both in close proximity to each other and in direct contact with the outermost oxide surfaces. This observation proved to be vital to the interpretation of the photocatalytic activity and bacterial attachment results as described in the following paragraphs.

The relative effect of different crystalline titanium oxide phases on the resulting PCA has been well documented in previous studies [19,49,50,51]. Generally, mixed phase oxides containing both anatase and rutile phases in close proximity to each other have resulted in higher PCA compared to single phase oxides [19,49,51]. Mixed phase oxides have been shown to trap electrons and holes, retard electron–hole recombination, and thus increase the duration of ROS activation [26]. Previous studies have also shown silver additions to improve the PCA compared to counterpart titanium oxides without silver [23,51,52,53]. Silver dopant incorporation has been shown to hinder the recombination of electrons and holes due to the presence of a Schottky barrier at the interface between titanium oxide and the silver [23,51,53].

In the present study, the C + Ag oxide, which was shown by XRD and EBSD analyses to be a predominately single phase rutile oxide, exhibited significantly higher PCA after 240 min of UVA illumination compared to the A + Ag silver-doped predominately anatase phase oxide. Furthermore, the C + Ag oxide also showed significantly higher PCA compared to its group C counterpart oxide with no silver doping. The B + Ag mixed phase oxides, also shown by XPS to take up the highest levels of silver doping, revealed statistically equivalent PCA levels compared to the C + Ag oxide. One potential explanation for this surprising result for the B + Ag silver-doped mixed phase oxide is that the phosphorus doping may have somewhat hindered the mixed phase PCA-enhancing effect. However, the reduction in PCA due to phosphorus-doping levels may have been somewhat counterbalanced by the higher silver-doping levels present in the B + Ag oxide to maintain an overall high PCA response.

Silver additions to titanium oxides have also shown an antibacterial effect [40,54]. Titanium nanotubes embedded with Ag_2_O nanoparticles showed 97–99% antibacterial efficacy against *E. coli* and *S. aureus* at day 24 of a previous study [40]. Silver deposition on pure titanium showed 99% antibacterial efficacy against *E. coli* and 90% antibacterial efficacy against *S. aureus* after 2 h in another previous study [54].

*S. aureus* was chosen for the bacterial testing in the present study due to its role as an early colonizer in the formation of biofilms and its prevalence in implant-related infections [5,55]. Specifically, *Staphylococci* species are a cause of up to four out of every five orthopedic implant infections [5]. Additionally, *S. aureus* possesses several cell surface adhesion molecules that help to facilitate attachment to both bone matrix and implant surfaces [5]. For the oxides kept under dark conditions in the present study, the silver-doped oxides did not show a significant change in the antibacterial effect compared to their non-silver counterparts. Among the silver-doped oxides, the B + Ag oxide showed significantly higher bacterial attachment compared to the A + Ag and C + Ag oxides. For the non-silver-containing oxides, the C oxide showed significantly lower levels of bacterial attachment compared to the B oxide. The higher bacterial attachment levels on the B and B + Ag oxides in this study may have also been the result of the additional phosphorus doping in these oxides.

Under UVA irradiation conditions, the B + Ag oxide exhibited significantly lower bacterial attachment compared to the B oxide counterpart without silver. This result supports the previous studies suggesting silver additions reduce bacterial attachment. However, XRD and EBSD analyses of the B + Ag oxide also revealed a mixed phase oxide with intermingled anatase and rutile phases near the outermost oxide surface. This type of mixed phase oxide has also been shown to enhance PCA and potentially the related bactericidal effect in previous studies [19,49,51]. Therefore, reduction in bacterial attachment shown for the B + Ag oxide in the present study was most likely a combination of increased silver-doping levels and the PCA-enhancing effect of the mixed phase oxide. Conversely, the higher bacterial attachment levels shown on the A + Ag and C + Ag oxides may be attributed to lower silver uptake as shown in the XPS analyses and single phase oxide layers as shown in the XRD and EBSD analyses.

The antibacterial activity of silver may be related to two different modes, including contact killing and ion-mediated killing or non-contact killing [56,57]. Contact killing takes place as a result of silver particles anchoring to the bacterial cell wall and infiltrating it, whereas non-contact killing arises from the release of silver ions which attack the bacteria [56,57]. In the present study, the primary bacterial reduction mechanism of the silver-doped oxides was likely contact killing, since the silver particles were incorporated into the oxide coatings. However, the ion-mediated killing mode from silver release may have also been active in this study. In the future we plan to evaluate the antibacterial activity of these coatings against other commonly acquired bacteria.

A small cell culture study was also performed to assess the cytocompatibility of the silver levels in the silver-doped oxides. Silver depositions up to 2.8% onto commercially sourced pure titanium discs through anodization have shown similar human fibroblast cytocompatibility compared to control titanium surfaces without silver deposition [33]. Titanium nanotubes embedded with 0.38–1.62 at % silver using TiAg magnetron sputtering showed almost no cytotoxicity to 3T3-E1 pre-osteoblasts after 5 days [40]. In the present study, silver uptake into the oxides ranged between 0.62 and 1.28 at %. The cytocompatibility of the silver-doped oxides was confirmed as shown in the MTT results in Figure 11. No significant differences in cell viability were shown between the silver-doped oxides and the non-modified CPTi surfaces on day 7 or 14. Among the silver-doped oxides, the A + Ag oxide exhibited significantly higher cell viability compared to the B + Ag and C + Ag oxides. This result may be attributed to the increased phosphorus uptake shown in the A + Ag oxide XPS dataset. Previous studies have shown improved osteoblast attachment and proliferation on phosphorus-doped titanium oxide surfaces compared to non-modified control titanium surfaces [13,58].

## 5. Conclusions

This study investigated the effects of silver doping additions to three established anodization processes. Silver doping was not shown to exhibit a significant influence on oxide crystallinity, surface topography, or surface wettability in the resulting oxides. Oxides formed using a sulfuric acid anodization process exhibited significantly enhanced PCA after silver doping, but silver-doped oxides formed in phosphoric-acid-containing anodization electrolytes showed no significant changes in PCA. *Staphylococcus aureus* attachment assays showed each oxide exhibited a photocatalytic antimicrobial effect as evidenced by a significant decrease in bacterial attachment under UVA irradiation. However, only the phosphorus-doped mixed anatase and rutile phase oxide exhibited an additional significant reduction in bacterial attachment under UVA irradiation as a result of silver doping. The antimicrobial success of this oxide was attributed to a combination of two previously successful strategies that are simultaneously possible through anodization processes. The formation of the mixed phase oxide was used to enhance the PCA-driven antimicrobial activity of the oxides and the silver-doping additions were utilized to chemically enhance the antimicrobial activity. The two-fold antimicrobial activity exhibited by these oxides shows much promise for future titanium implant coatings.

## Figures and Tables

**Table 1:** Electrolytes used for each anodization process.

Oxide Groups	Sulfuric Acid (M)	Phosphoric Acid (M)	Hydrogen Peroxide (M)	Oxalic Acid (M)	Silver Nitrate ^1^ (M)
A	3.5	0.19	0.75	0.25	-
A + Ag	3.5	0.19	0.75	0.25	0.077
B	1.4	0.03	0.75	-	-
B + Ag	1.4	0.03	0.75	-	0.059
C	1.0	-	-	-	-
C + Ag	1.0	-	-	-	0.071

^1^ The silver nitrate concentrations listed represent the saturation limit within the mixed-acid electrolytes.

**Table 2:** Surface roughness values for each oxide (mean ± SD).

Oxide Groups	R_a_ (nm)	R_z_ (µm)
A	314.4 ± 13.6	2.9 ± 0.1
A + Ag	291.2 ± 3.9	3.3 ± 0.2
B	289.3 ± 13.8	2.7 ± 0.3
B + Ag	291.2 ± 38.9	3.1 ± 0.5
C	410 ± 109	4.1 ± 1
C + Ag	254.8 ± 48.6	2.9 ± 1.1

**Table 3:** Oxide group surface chemistries (mean ± SD).

Oxide Groups	Titanium (at %)	Oxygen (at %)	Carbon (at %)	Sulfur (at %)	Silver (at %)	Phosphorus (at %)
A	22.18 ± 0.43	55.35 ± 0.19	18.2 ± 0.23	0.50 ± 0.08	-	3.64 ± 0.12
A + Ag	20.14 ± 0.74	53.83 ± 1.51	21.14 ± 2.00	0.96 ± 0.87	0.80 ± 0.09	3.14 ± 0.23
B	23.00 ± 1.16	54.72 ± 1.41	18.71 ± 2.44	0.84 ± 0.27	-	2.73 ± 0.11
B + Ag	22.19 ± 0.65	54.06 ± 0.51	19.35 ± 0.94	0.46 ± 0.01	1.28 ± 0.05	2.65 ± 0.15
C	17.77 ± 2.94	45.00 ± 1.67	33.22 ± 2.42	1.77 ± 1.30	-	-
C + Ag	22.23 ± 0.35	49.76 ± 0.40	25.73 ± 0.60	1.56 ± 0.03	0.61 ± 0.03	-

## Data Availability

The data that support the findings of this study are available from the corresponding author upon reasonable request.
